# Clinical, Dermoscopic and Histopathological Features of Non-melanoma Skin Cancers in People With Skin of Colour: A Series of Five Cases

**DOI:** 10.7759/cureus.61192

**Published:** 2024-05-27

**Authors:** Guntamukkala Geeta Sai, Sheba Mariam Jacob, Sai Kavya D, Anannya S, Afthab Jameela Wahab, Vimal Chander R

**Affiliations:** 1 Department of Dermatology, Saveetha Medical College and Hospital, Saveetha Institute of Medical and Technical Sciences (SIMATS) Saveetha University, Chennai, IND; 2 Department of Pathology, Saveetha Medical College and Hospital, Saveetha Institute of Medical and Technical Sciences (SIMATS) Saveetha University, Chennai, IND

**Keywords:** case series, dermatology, nonmelanoma skin cancer, squamous cell carcinoma, basal cell carcinoma

## Abstract

Non-melanoma skin cancers (NMSC) such as basal cell carcinoma (BCC) as well as squamous cell carcinoma (SCC) are the two most common skin malignancies globally. They are observed more frequently among Caucasians than Asians, and their incidence is inversely proportional to the pigmentation levels. Even though the occurrence of skin cancers in India is lower, the absolute quantity of cases may be considerable due to the vast population. Here, we report five cases of NMSC in people having skin of colour.

## Introduction

Skin cancers, in general, comprise cutaneous melanoma (CM), and the two most common types of non-melanoma skin cancers (NMSCs) are squamous cell carcinoma (SCC) and basal cell carcinoma (BCC). Malignant skin tumors make up 20-30% of all cancers in Caucasians, compared to 2-4% in Asians, and 1-2% in African Americans and Indians [1]. Dark-skinned individuals naturally produce more melanin and possess larger, more dispersed melanosomes, filtering double the ultraviolet radiation as compared to the smaller, clustered melanosomes found in Caucasian skin. This natural mechanism provides increased protection against cutaneous malignancies. BCC and SCC account for 99% of all NMSCs [2].

Both BCC and SCC arise from epidermal keratinocytes. Other NMSCs include sebaceous carcinoma, Merkel cell carcinoma, apocrine adenocarcinoma and other rare tumors [3,4]. SCC is the predominant type of cutaneous neoplasm, accounting for 30-65% of cases among African Americans and Indians, whereas it is the second most prevalent after BCC in individuals with white skin, comprising 15-25% of cutaneous malignancies [1]. The ratio of BCC and SCC varies between 1:1 and 10:1 based on population, ethnicity, and gender [5,6]. Mathematical models suggest that with each 1% reduction in the ozone layer, there is a 2-4% rise in tumor incidence, with the increase being more significant for SCC than for BCC [7]. When people of colour get skin cancer, their prognosis is typically worse than that of Caucasian patients because they usually present with an advanced stage of the disease. For patients with darker skin, clinicians should be aware that darker pigmentation is a common feature of basal cell carcinoma (BCC) and can clinically resemble seborrheic keratosis or melanoma. Therefore, evaluation with dermoscopy is warranted. 

The dermoscopic features associated with basal cell carcinomas include, the absence of a pigment network and the presence of specific features such as large blue-gray ovoid nests, arborizing vessels, maple leaf-like areas, spoke wheel areas, multiple blue-gray globules, and ulceration. Maple leaf-like and spoke-wheel areas, blue-grey ovoid nests are features associated with melanin [8]. Additionally, vascular patterns such as short fine telangiectasias (SFTs), milky-pink backgrounds and arborizing microvessels have been reported, and these patterns may be particularly useful for identifying non-pigmented BCCs [9]. Dermoscopy criteria for squamous cell carcinoma includes the presence of keratin/scales, white structureless areas, hairpin vessels, blood spots, perivascular white halos, linear-irregular vessels, and ulceration. Keratin and scales appear as homogeneous, opaque yellow to brown structures, representing hyperkeratosis and parakeratosis. Blood spots, which are multiple red to black dots within the keratin mass, correspond to small crusts or hemangiomas [10]. Here, we report five cases of NMSC in people with skin of colour.

## Case presentation

Case 1

**Figure 1:**
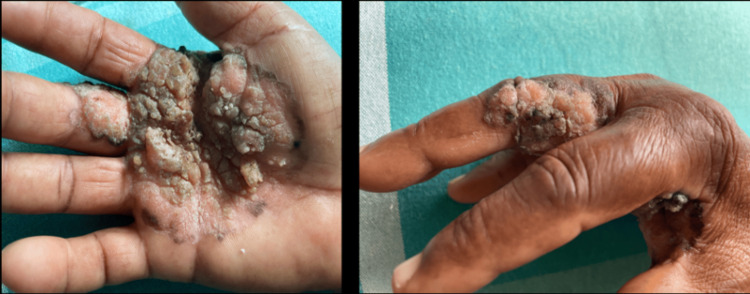
Well-defined verrucous exophytic plaque with erosions and surrounding hyperpigmented margin over the right palm and middle finger

62-year-old female presented with rough raised skin lesions over her right palm and middle finger, which had been present for eight years. The lesions were associated with itching and pain for 4 weeks. The initial lesion started as a small asymptomatic dark coloured skin lesion on the right palm, gradually increasing in size over time without any antecedent trauma. Upon examination, a single well-defined verrucous exophytic plaque measuring 10x10 cm with erosions and surrounding hyperpigmented margin was observed over the right palm and middle finger (Figure 1).

**Figure 2:**
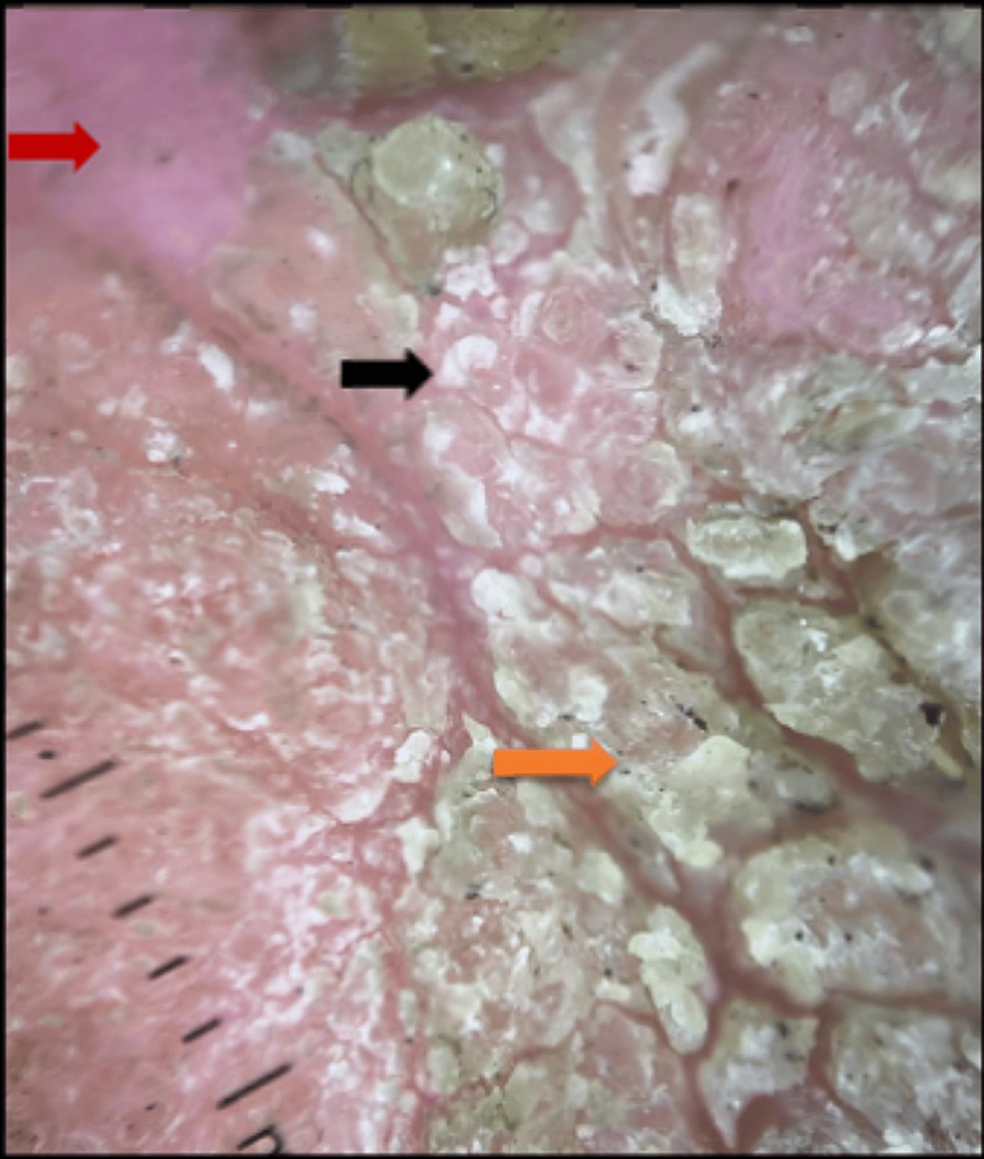
Dermoscopy showing keratin mass (orange arrow), surface scaling (black arrow), white pink structureless area (red arrow).

Clinical differential diagnoses were verrucous carcinoma, Bowen's disease, tuberculosis verrucosa cutis, chromoblastomycosis, and keratotic BCC. Dermoscopy revealed keratin mass and surface scaling, a white-pink structureless area with no vascular morphology (Figure 2).

**Figure 3:**
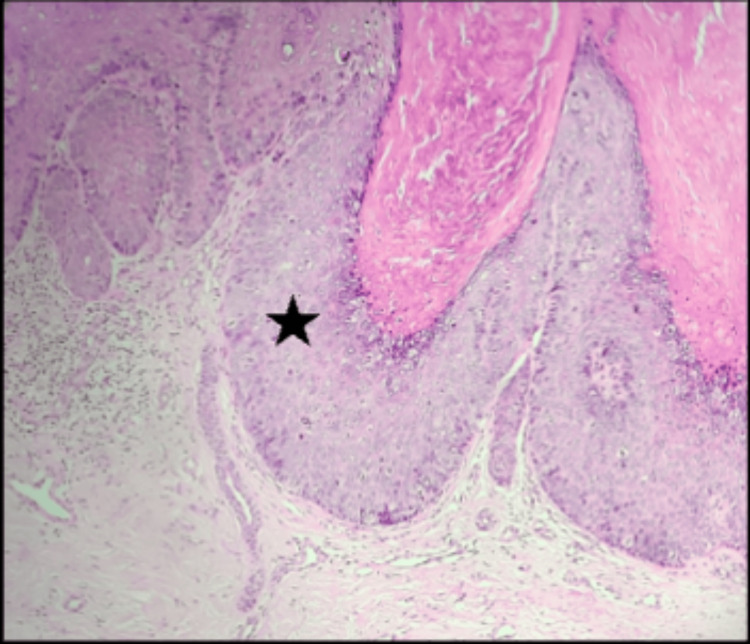
Tissue section stained with hematoxylin and eosin (10X magnification) showing hyperkeratosis, acanthosis, vacuolated keratinocytes, full thickness loss of polarity (black star).

Histopathological analysis revealed hyperkeratosis, acanthosis, complete thickness loss of polarity (wind-blown appearance), variation in cell size, and vacuolated keratinocytes (Figure 3), consistent with Bowen's disease.

**Figure 4:**
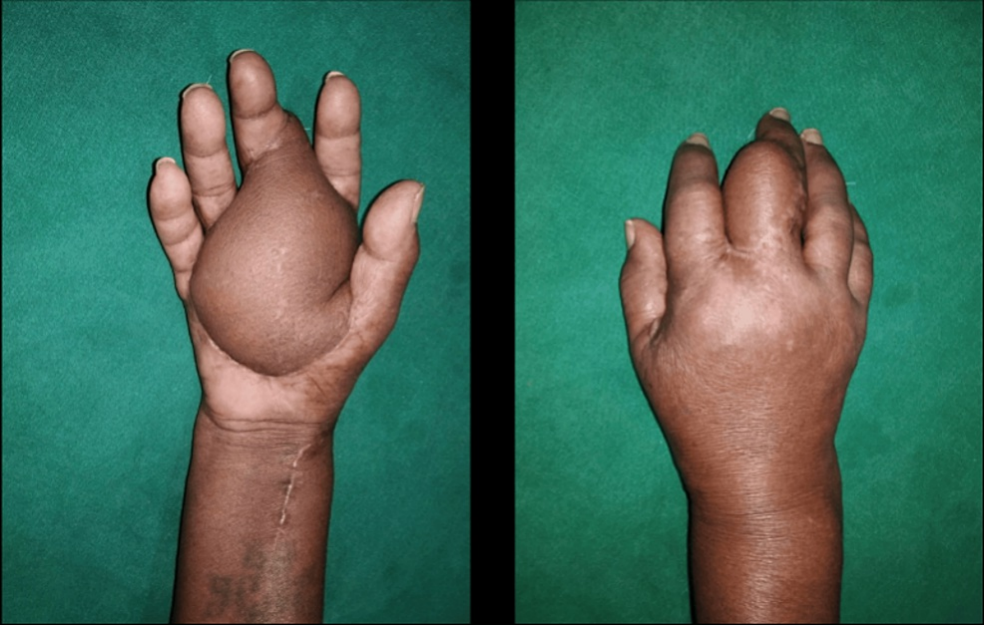
Post operative image.

Further investigations, including potassium hydroxide (KOH) mount, fungal culture and sensitivity, Mantoux test, Gram staining, and AFB staining, yielded negative results. X-ray of the hand was normal. The patient underwent wide local excision of the entire lesion, followed by a free anterolateral thigh flap (Figure 4).

Case 2

**Figure 5:**
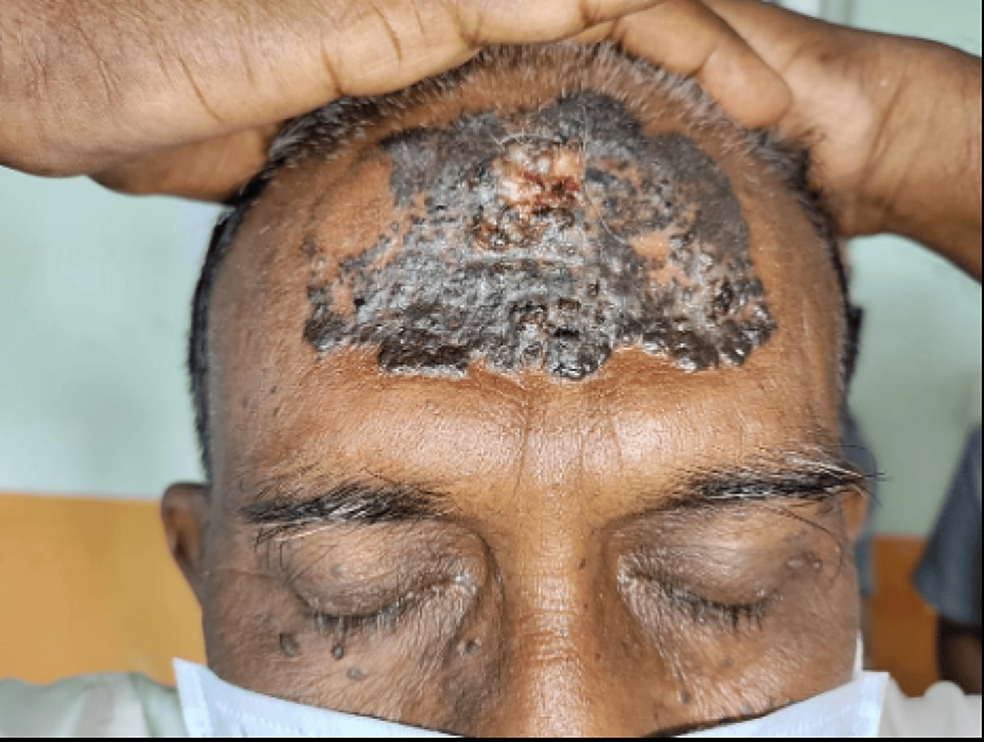
Well defined hyperpigmented plaque with central ulceration and rolled margins over the forehead.

60-year-old male patient presented with a history of multiple dark raised skin lesions over the face for 25 years, with an increase in size and ulceration over one of the forehead lesions for five years. The initial lesion started as a small, dark, raised, asymptomatic skin lesion on the forehead, gradually increasing in size over time with ulceration. Upon examination, a single well-defined hyperpigmented plaque measuring 6x8 cm with central ulceration and rolled margins was noted over the forehead (Figure 5).

**Figure 6:**
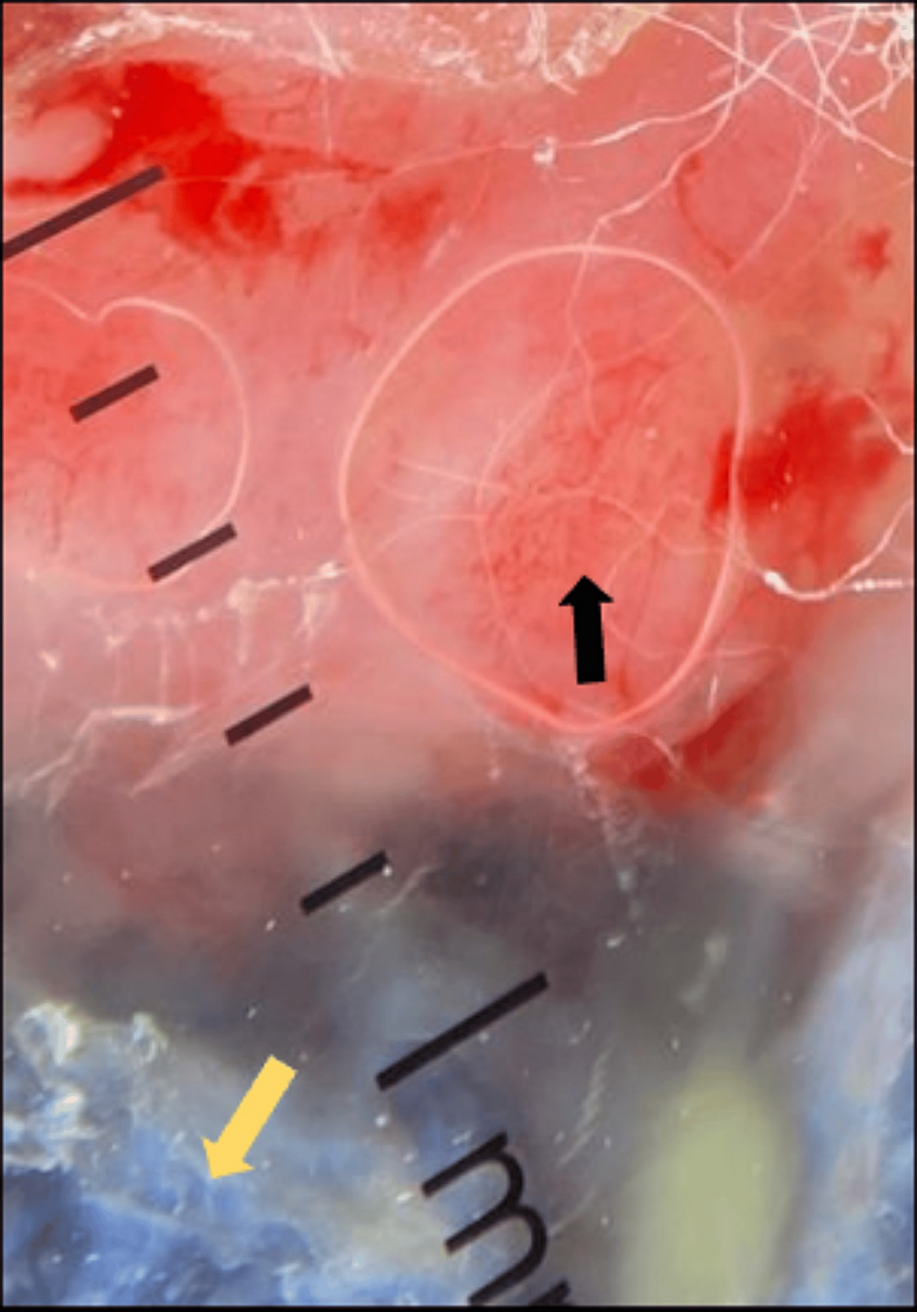
Dermoscopy showing arborizing blood vessels (black arrow), blue grey areas (yellow arrow).

 Dermoscopy revealed arborizing blood vessels, blue-grey areas, and keratotic scales (Figure 6).

**Figure 7:**
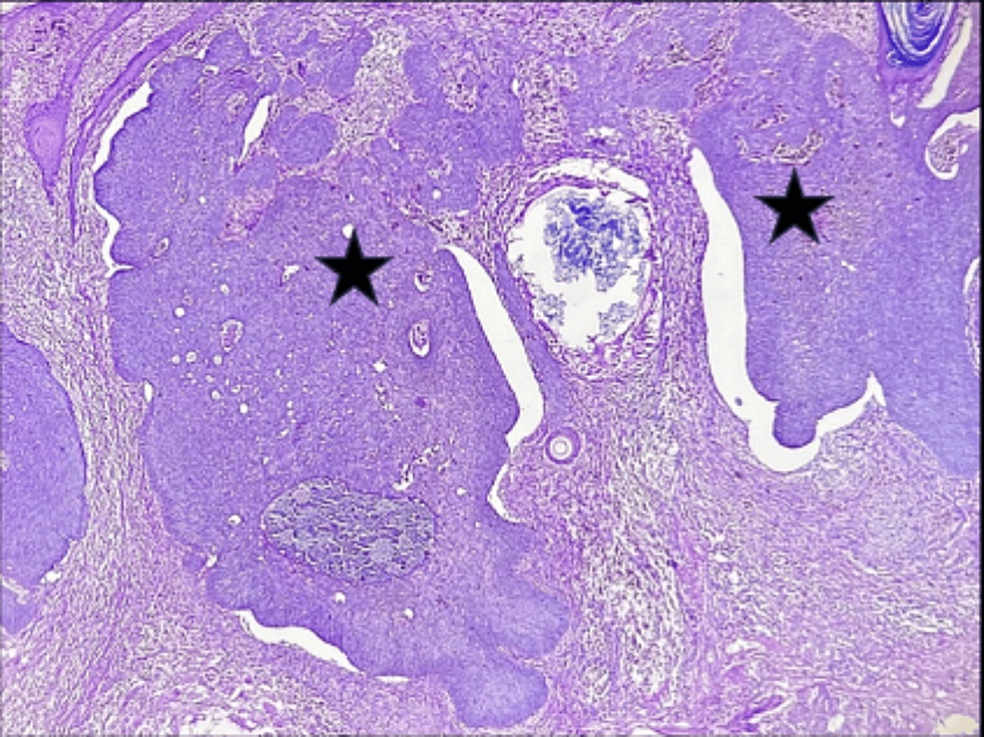
Tissue section stained with hematoxylin and eosin (10X magnification) showing basaloid cell nests with peripheral palisading pattern (black star).

Histopathology revealed features consistent with basal cell carcinoma with basaloid cell nests showing peripheral palisading patterns (Figure 7).

**Figure 8:**
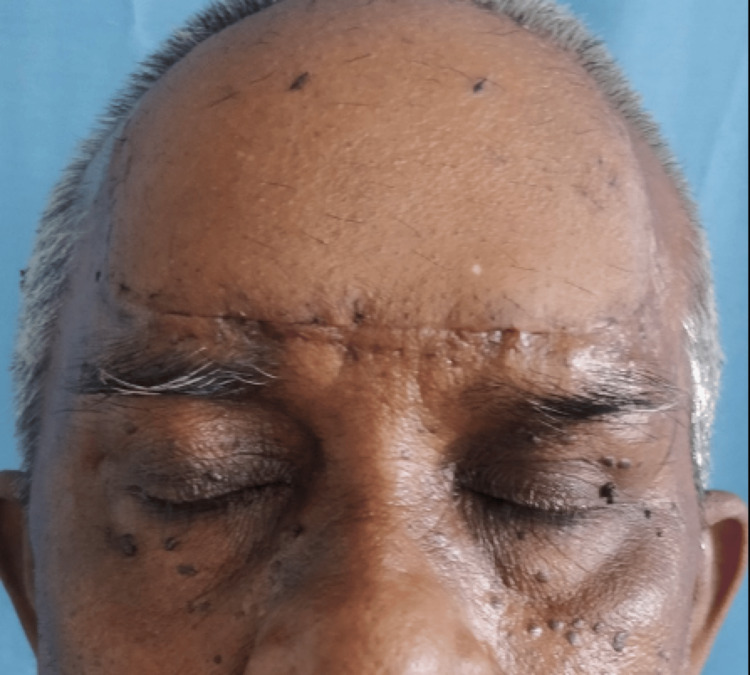
Post operative image.

The patient was diagnosed with basal cell carcinoma with seborrheic keratosis. He underwent wide local excision of the entire lesion, followed by secondary skin grafting (Figure 8).

Case 3

**Figure 9:**
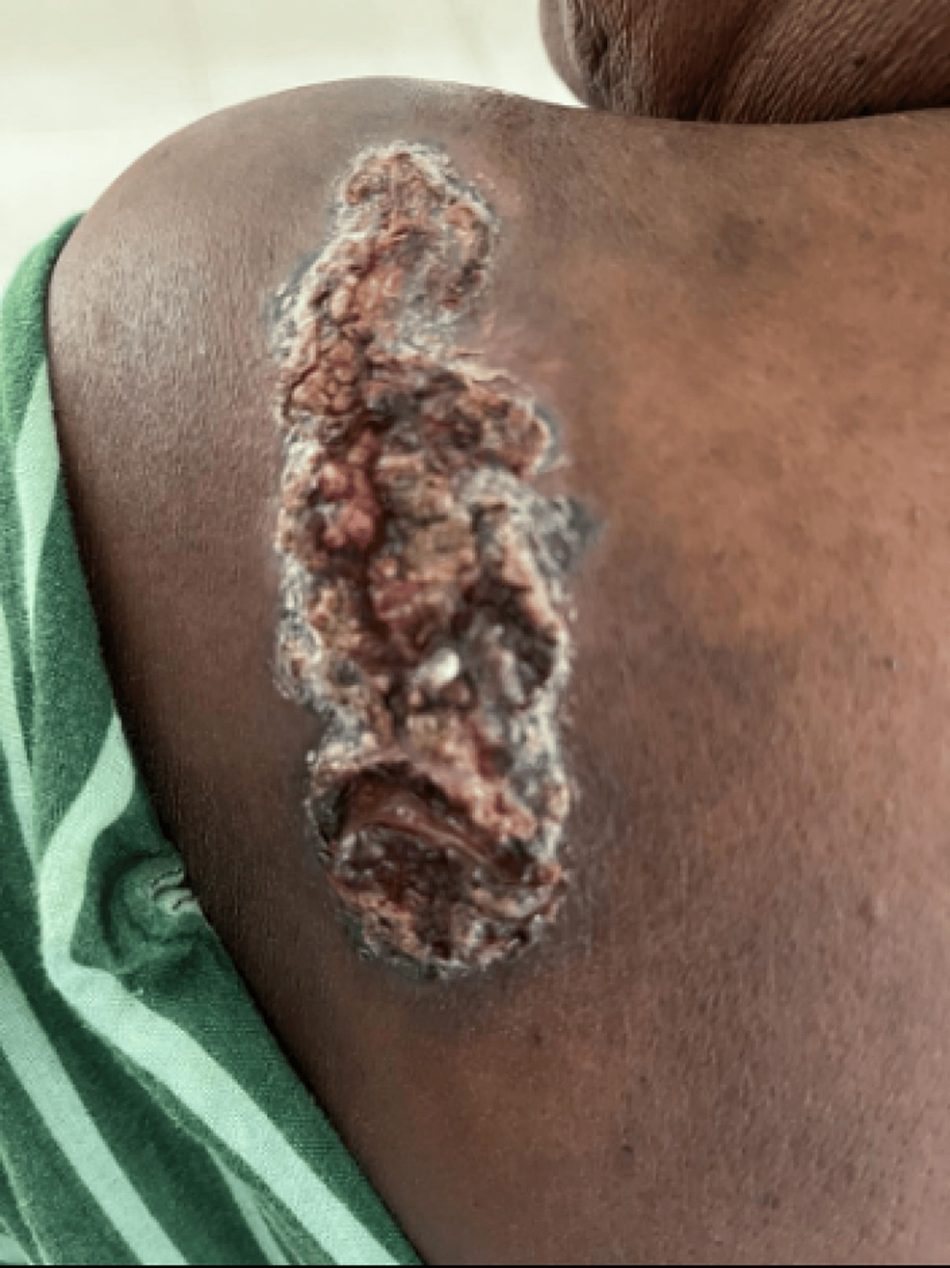
Well defined hyperpigmented plaque with crusting over the left shoulder and back.

57-year-old female patient presented with a dark, rough, raised skin lesion over the left shoulder for 15 years. The lesion started as a small, dark, raised skin lesion, which gradually increased in size with bloody discharge and crusting for three years. On examination, a well-defined hyperpigmented plaque measuring 8x3 cm with crusting was noted over the left shoulder and the back (Figure 9).

**Figure 10:**
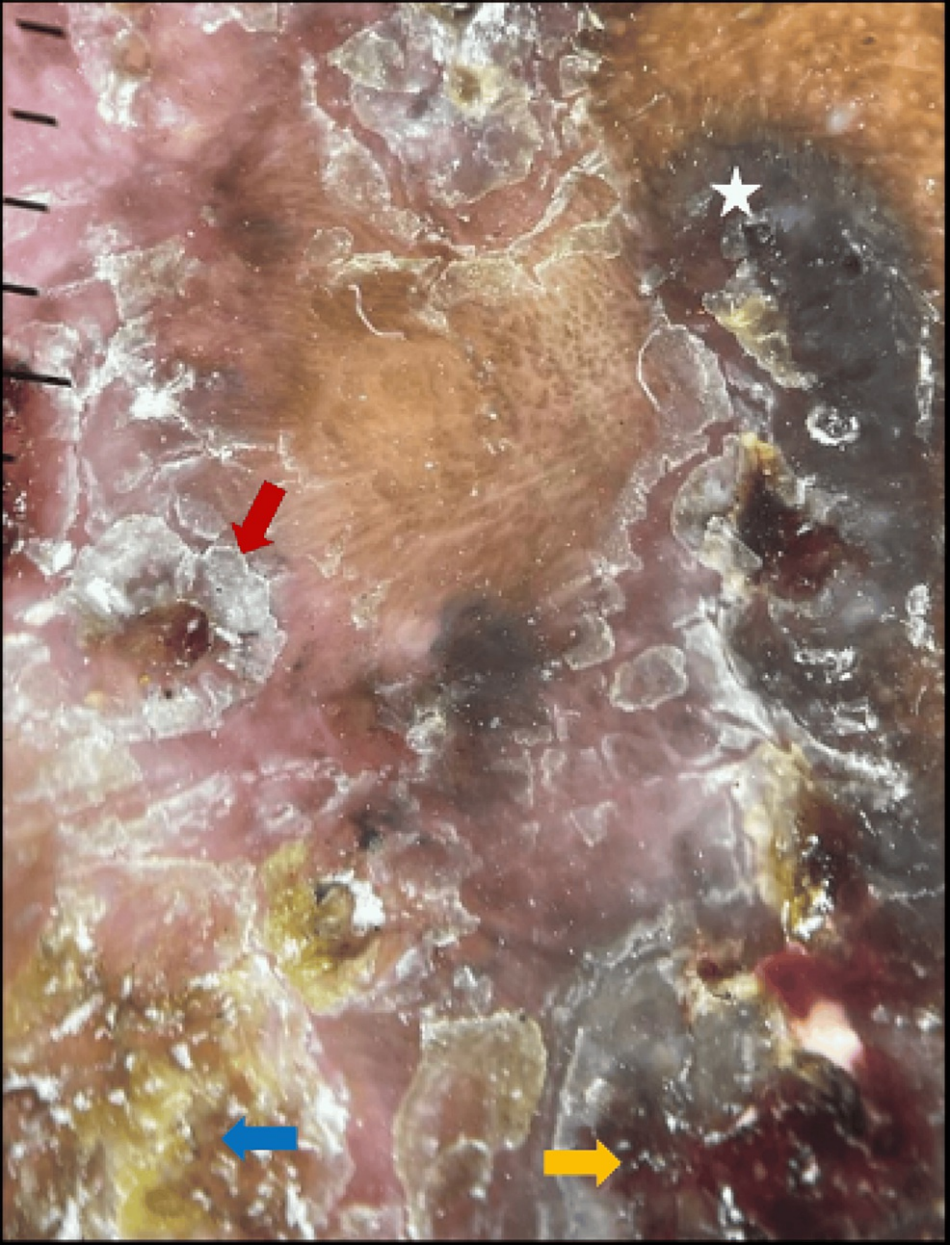
Dermoscopy showing keratin mass (blue arrow), surface scaling (red arrow), erosions (yellow arrow), blue grey structureless area (white star).

Dermoscopy showed keratin mass, surface scaling, blue-grey structureless areas, and erosions (Figure 10).

**Figure 11:**
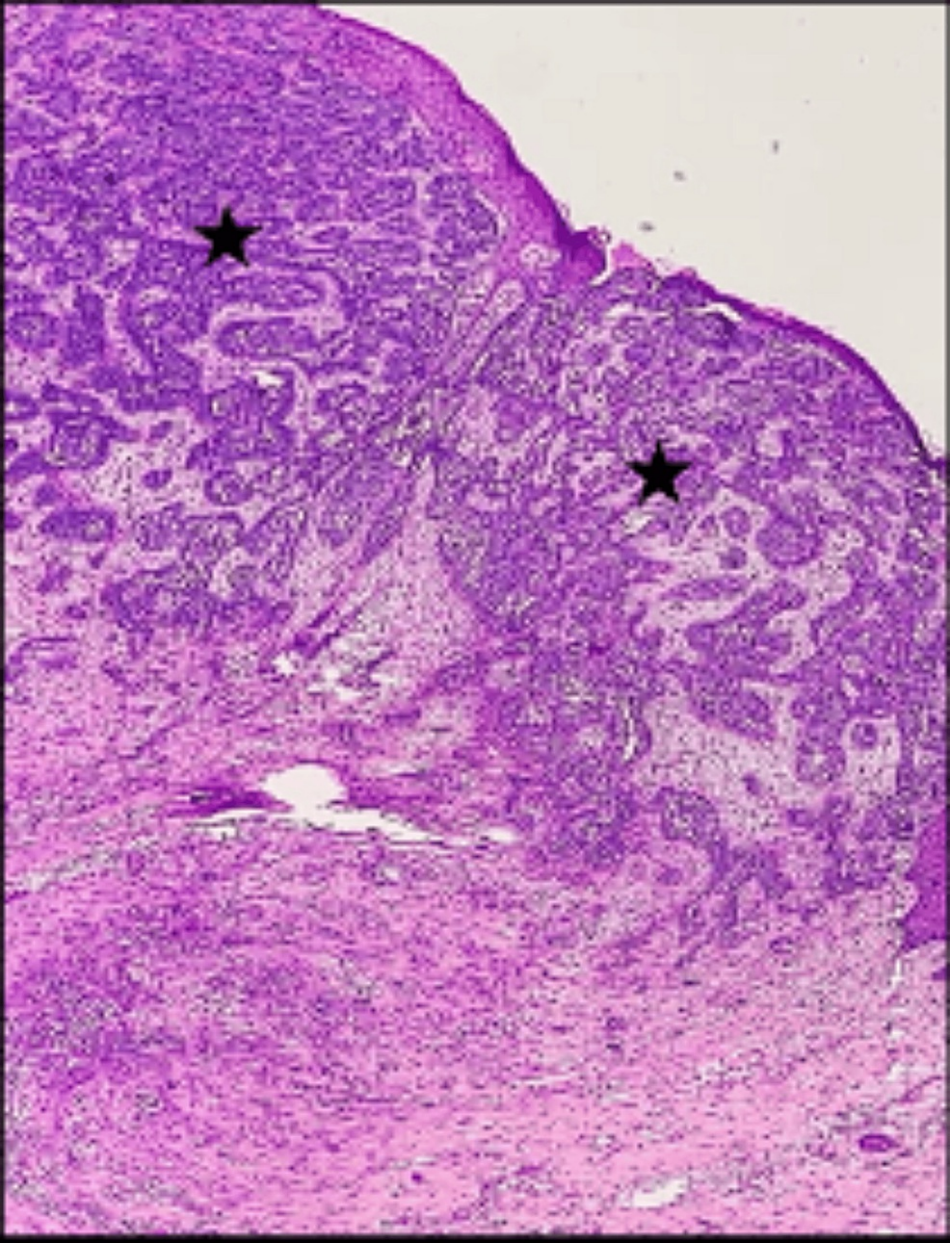
Tissue section stained with hematoxylin and eosin (10X magnification) illustrating malignant neoplasm composed of basaloid cell nests (black star).

**Figure 12:**
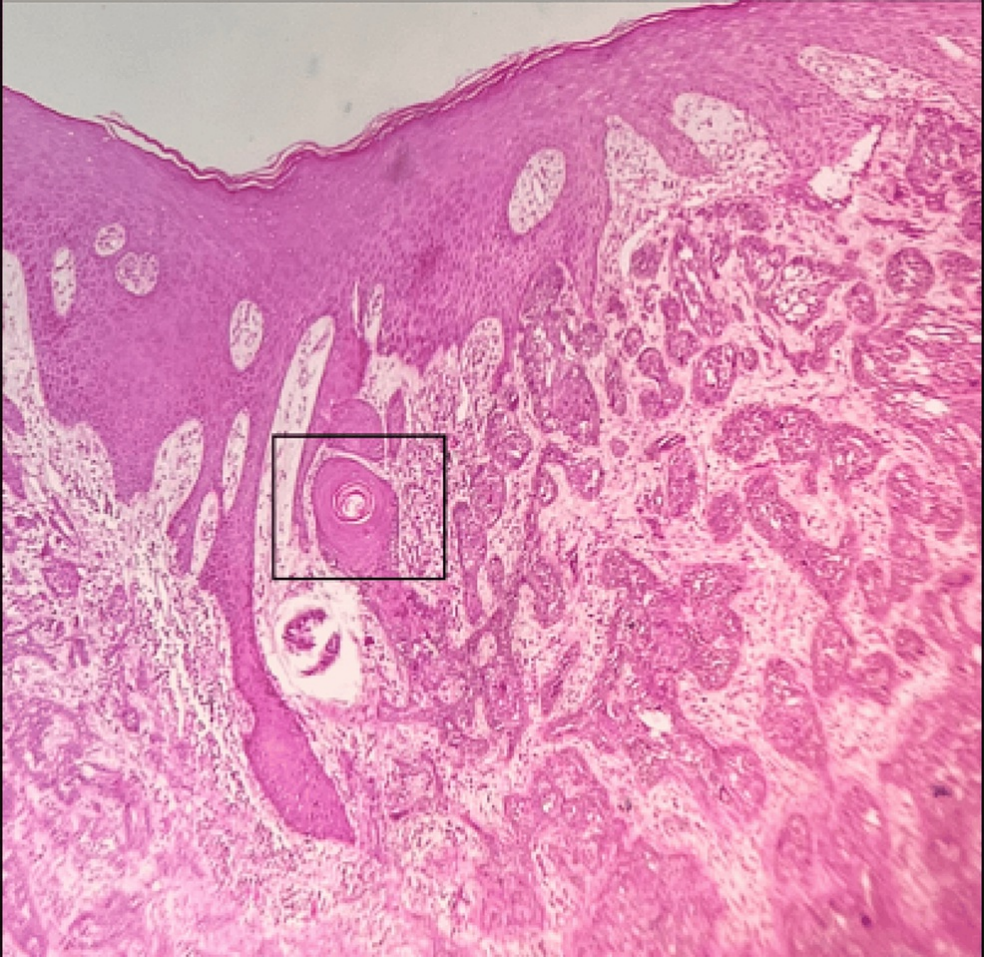
Tissue section stained with hematoxylin and eosin (10X magnification) illustrating focal keratin pearl.

Skin biopsy and histopathological examination revealed malignant neoplasm composed of cell nests with peripheral palisading basaloid cells (Figure 11) and retraction artifacts with focal keratin pearl formation (Figure 12).

**Figure 13:**
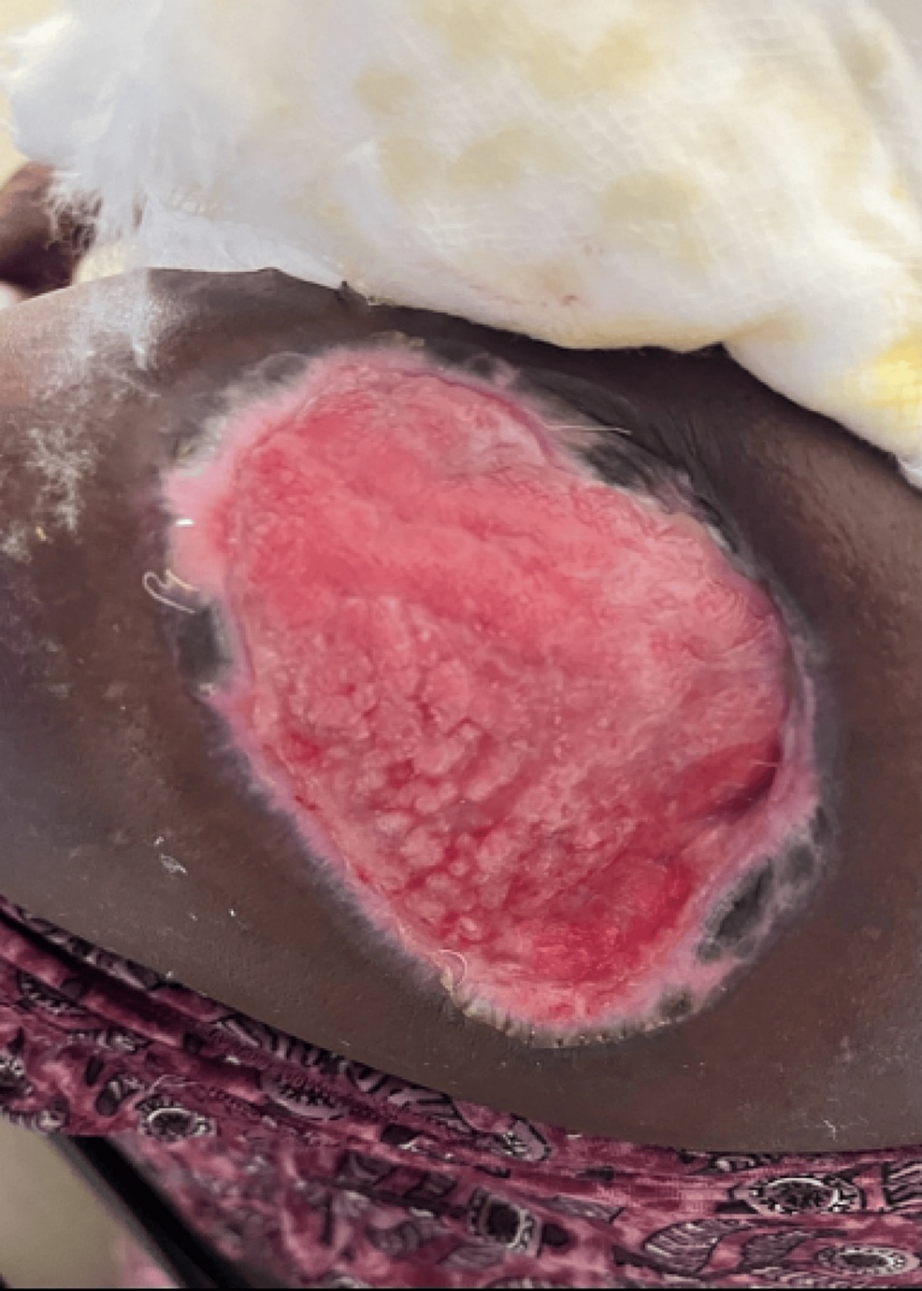
Post operative image showing graft rejection.

The patient was diagnosed with basosquamous carcinoma. She underwent wide local excision of the entire lesion, followed by secondary skin grafting, but unfortunately experienced graft rejection (Figure 13).

Case 4

**Figure 14:**
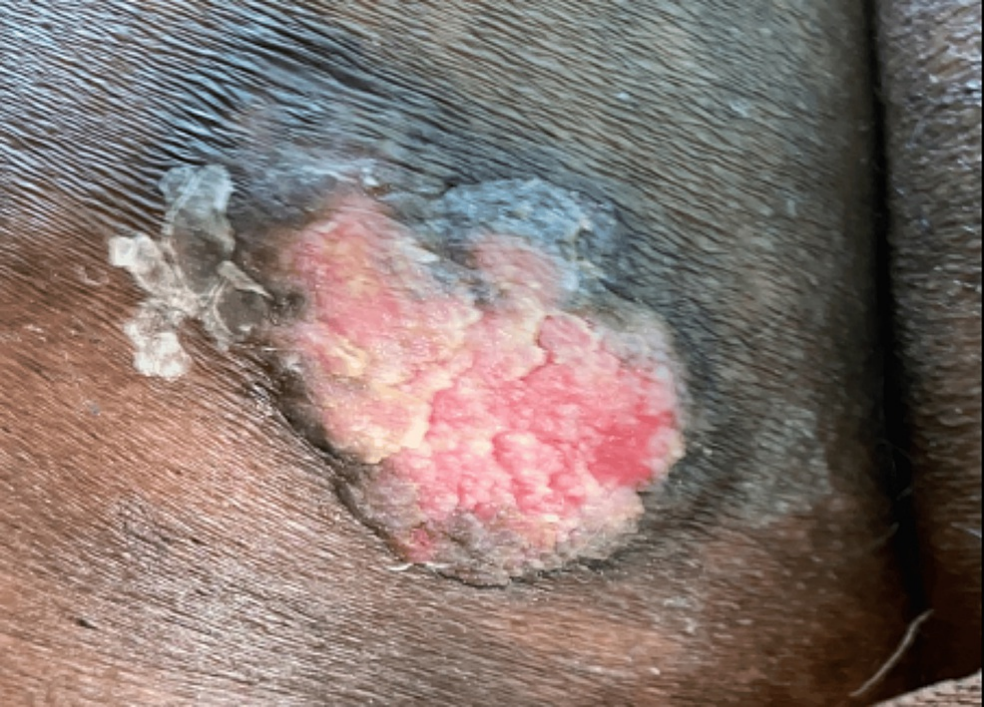
Single erythematous verrucous plaque over the left gluteal region.

70-year-old male patient presented with a rough raised skin lesion over the left side of the buttocks for five years. The lesion started as an asymptomatic small red raised skin lesion that gradually increased in size. On examination, a single erythematous verrucous plaque measuring 5x4 cm was noted over the left gluteal region (Figure 14).

**Figure 15:**
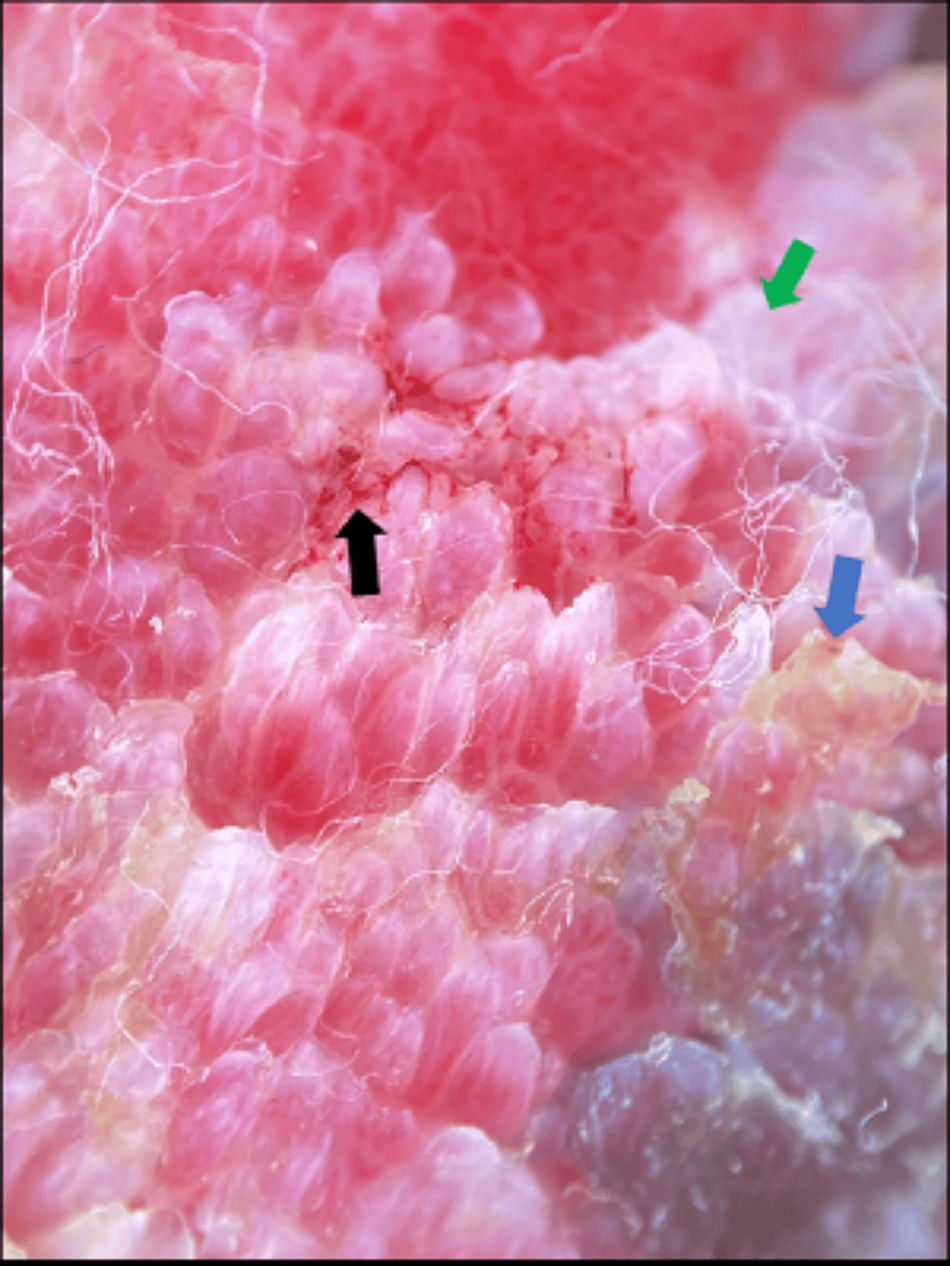
Dermoscopy showing surface keratin (blue arrow), white structureless area (green arrow), multiple branched blood vessels (black arrow).

Dermoscopy revealed surface keratin, multiple branched blood vessels, and ulceration (Figure 15).

**Figure 16:**
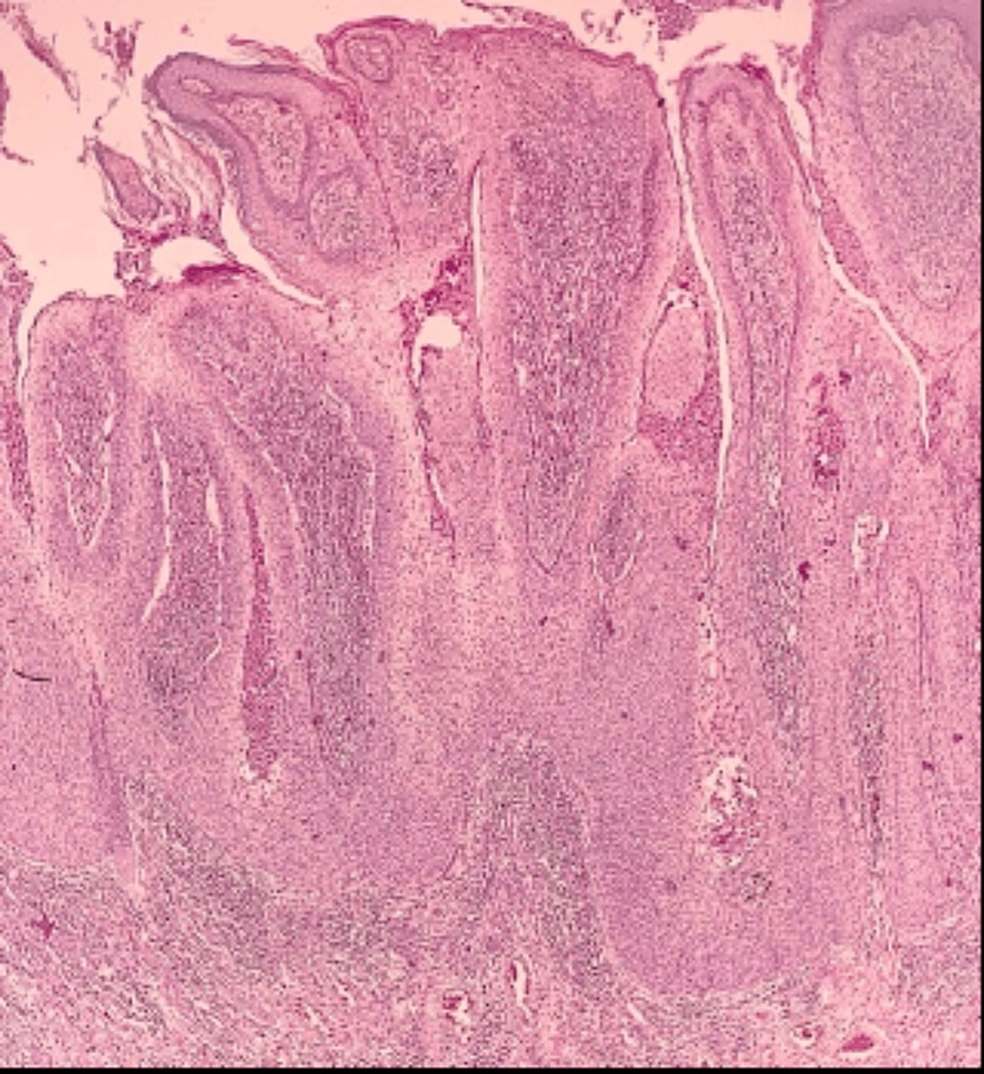
Tissue section stained with hematoxylin and eosin (10x magnification) illustrating anastomosing bundles and nests of polygonal cells with pleomorphic hyperchromatic nuclei with a pushing border.

Skin biopsy and histopathology revealed tumors arranged in anastomosing bundles and nests of polygonal cells with pleomorphic hyperchromatic nuclei with a pushing border (Figure 16).

**Figure 17:**
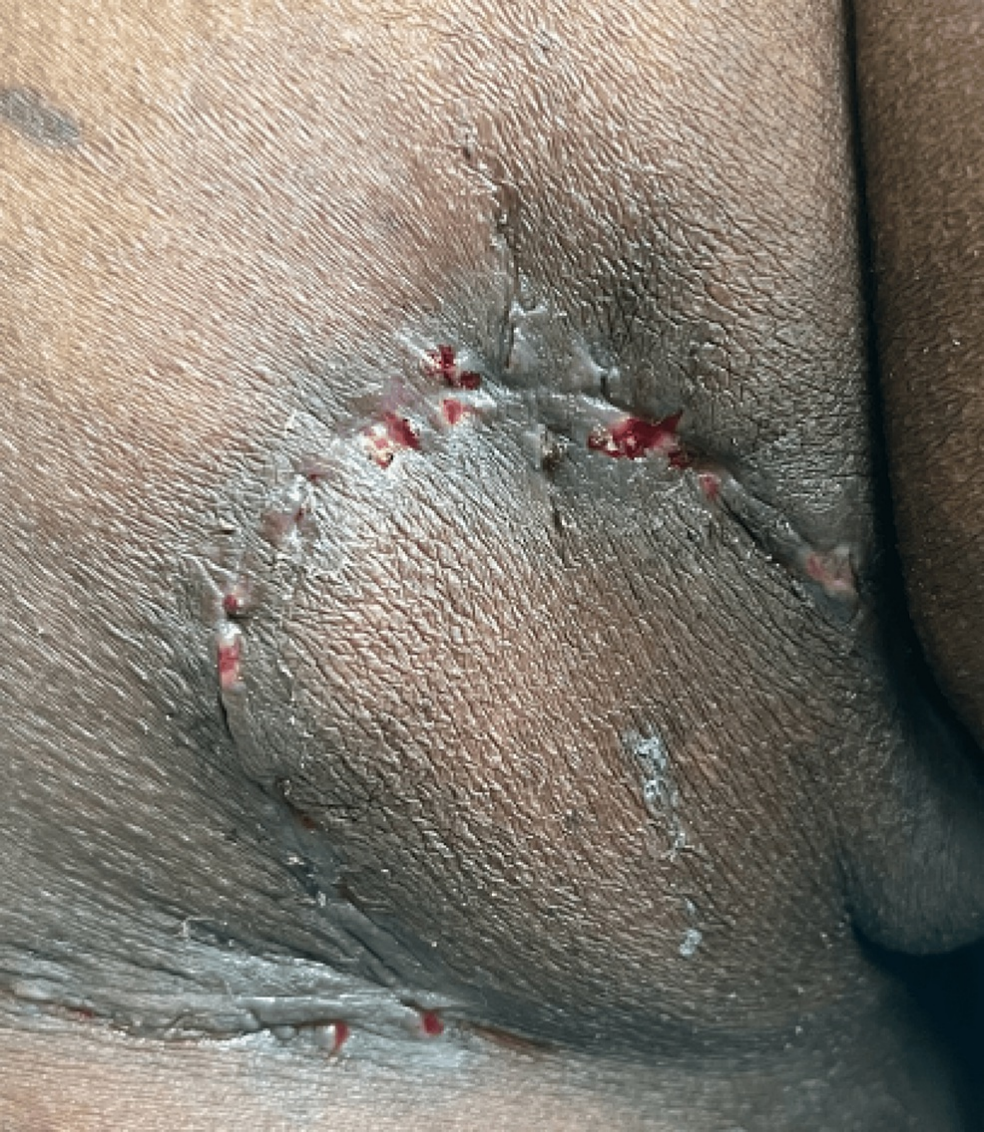
Post operative image

He was diagnosed with verrucous SCC. The patient underwent wide local excision of the entire lesion, followed by secondary skin grafting (Figure 17).

Case 5

**Figure 18:**
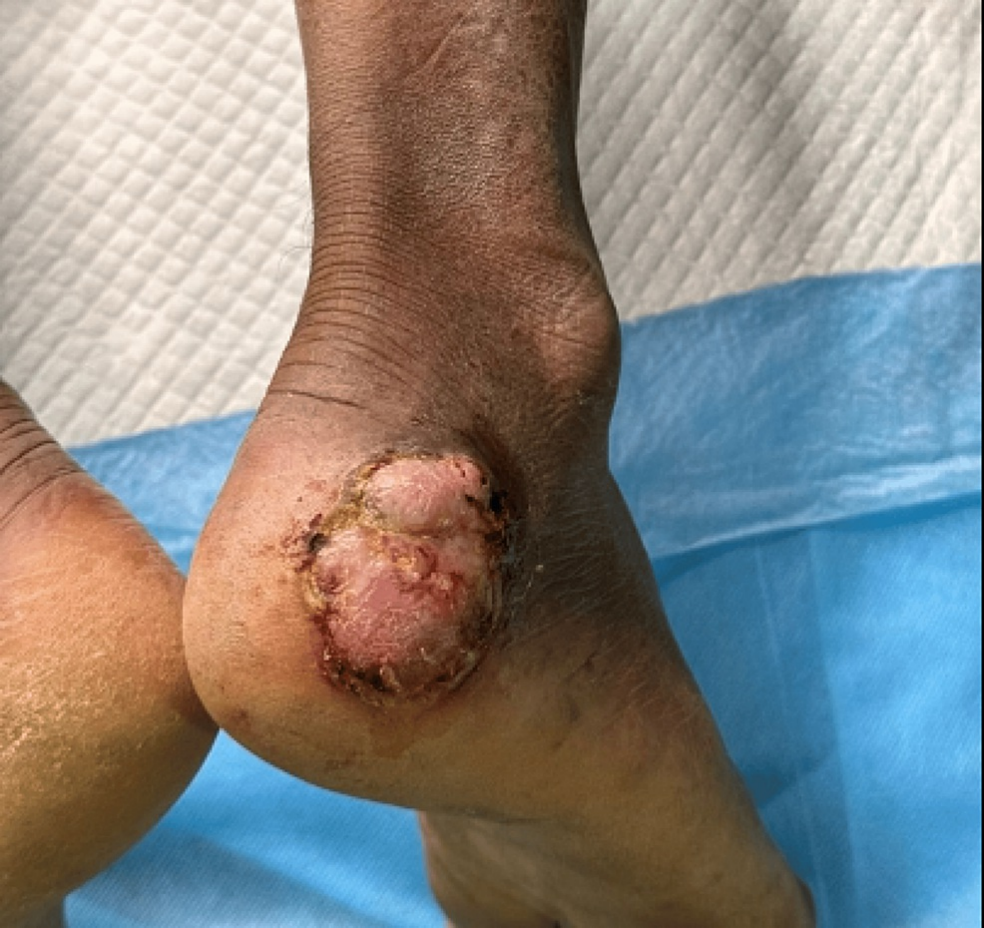
Well defined exophytic growth with ulceration, bloody discharge from few areas, crusting over posterolateral aspect of right foot

60-year-old male patient presented with raised skin lesions over the right foot for one and a half years, with crusting and oozing over the lesion for six months. On examination, a single well-defined exophytic growth measuring 5x6 cm with ulceration, bloody discharge from a few areas, and crusting was noted over the posterolateral aspect of the right foot (Figure 18).

**Figure 19:**
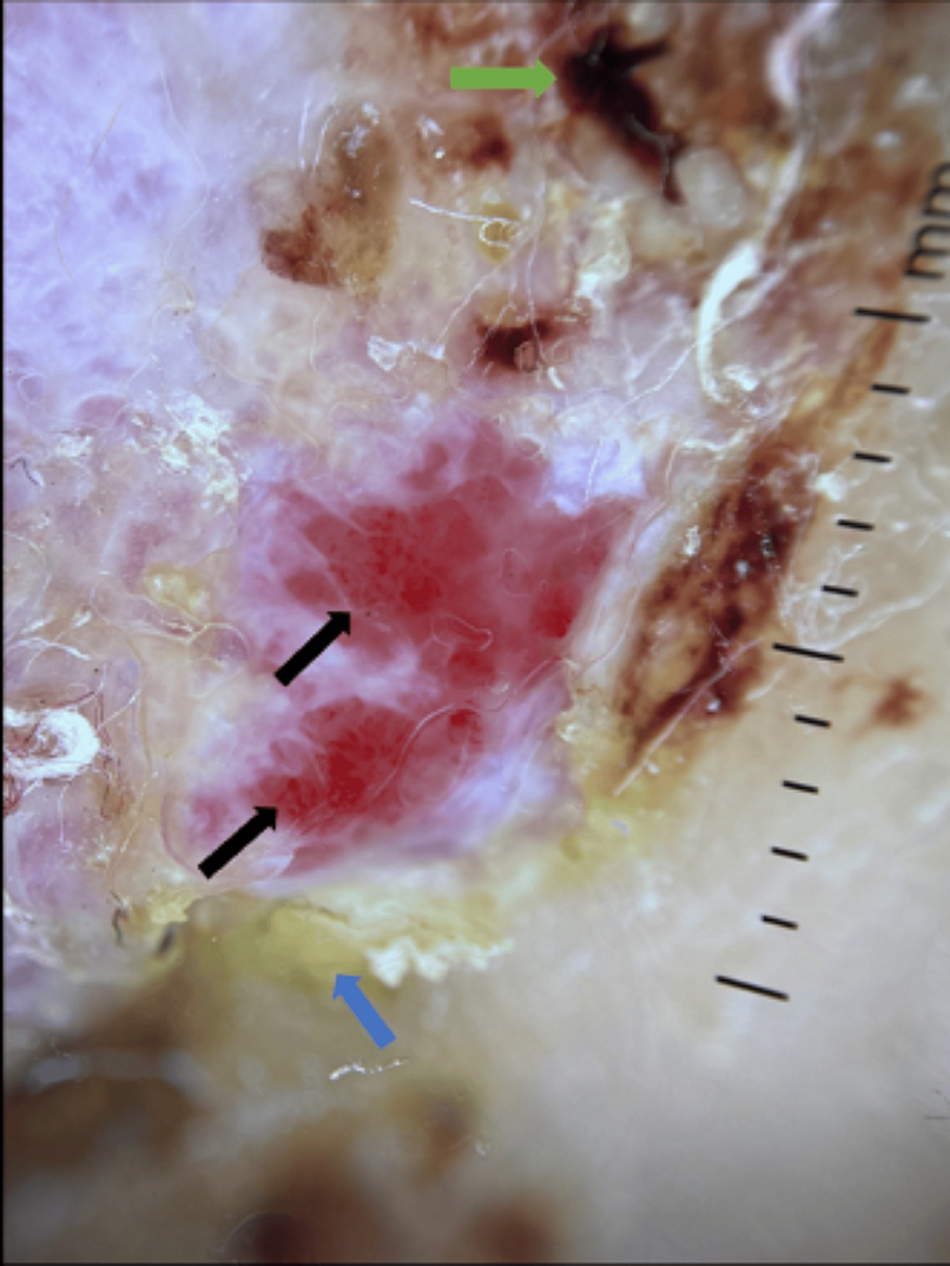
Dermoscopy showing dotted and coma shaped vessels (black arrow), keratin mass (blue arrow), erosions (green arrow).

Dermoscopic examination revealed dotted and coma-shaped vessels, keratin mass, and erosions (Figure 19).

**Figure 20:**
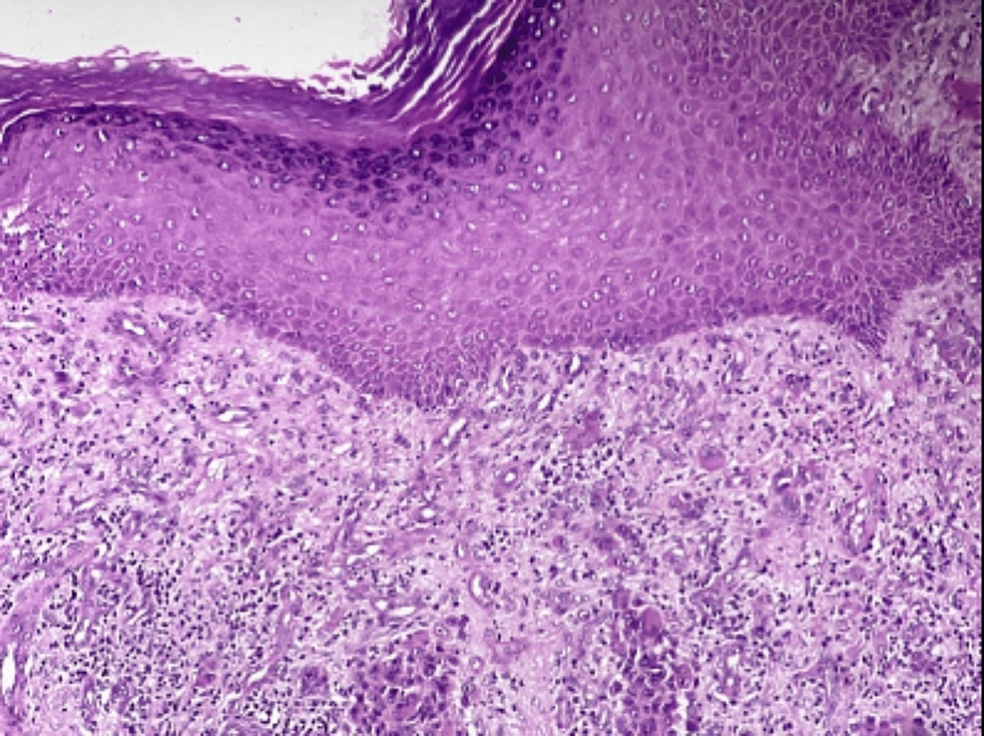
Tissue section stained with hematoxylin and eosin (100X magnification) showing dermis with irregular nests and sheets of malignant squamous cells.

Skin biopsy was taken, and histopathological examination revealed extensive hyperkeratosis, acanthosis, irregular elongation of rete ridges, focal areas of ulceration, dermis showing irregular nests and sheets of malignant squamous cells with moderate chronic inflammatory cell infiltrate (Figure 20).

**Figure 21:**
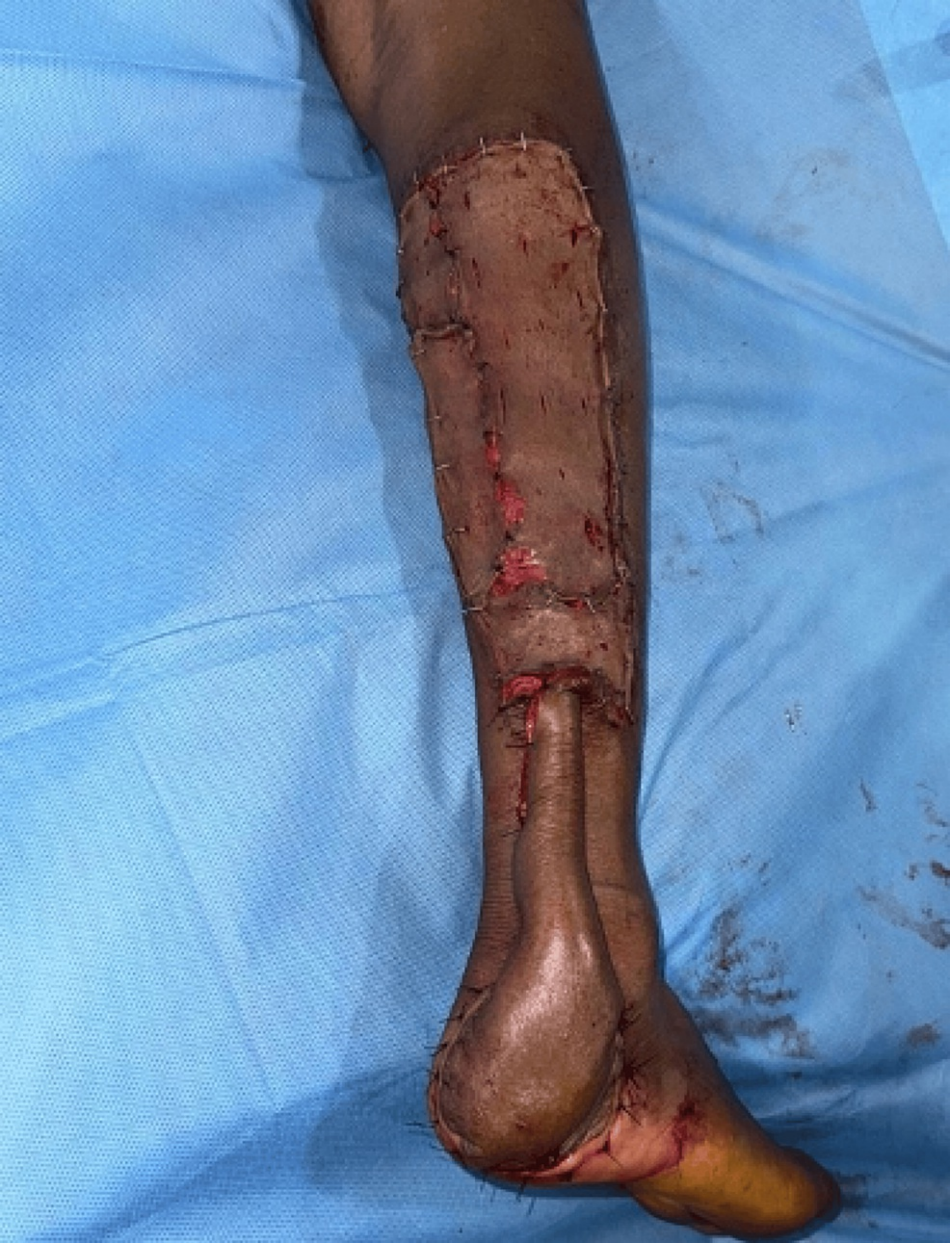
Post operative image

He was diagnosed with squamous cell carcinoma and underwent right foot reverse superficial sural artery flap with a split-thickness skin graft (Figure 21).

## Discussion

We report a series of five cases of non-melanoma skin cancers in skin of colour patients. UVB radiation, with a wavelength range between 290 and 320 nm, are the most significant wavelengths for developing cutaneous malignancies, according to experimental and epidemiologic data [11]. The risk of cutaneous malignancies rises due to increased UVB radiation reaching the earth's surface because of the depletion of the ozone layer. Additional noteworthy causative elements comprise arsenic, coal tar, radiation therapy, and different types of hydrocarbons [12]. BCC is the most common carcinoma that develops in the body region receiving radiotherapy [13].

Human papillomavirus (HPV) might impact immunocompromised patients [14], especially in SCC. Immunocompromised patients, particularly those who underwent organ transplantation, are also prone to develop these tumors [15]. The exact incidence in India remains unknown. However, non-melanoma skin çancer is considered rare among Asians [16]. The majority of SCCs arise in sun-exposed areas [17]. Occasionally non-exposed areas, not typically exposed to the sun, are affected particularly in individuals with heavily pigmented skin [18]. SCCs are predominantly observed among older individuals and are uncommon in childhood and adolescence [19]. Clinically, they appear as shallow ulcers that frequently have elevated, indurated margins and a keratinized crust. The morphological variations of SCC involve Bowen's disease, verrucous carcinoma, giant condyloma, keratoacanthoma, bowenoid papulosis [20]. Sunlight and exposure to arsenic are commonly implicated in the development of Bowen's disease [21].

BCCs are predominantly found on sun-exposed areas of the skin and are less prevalent among individuals with darker skin tones [22,23]. Approximately 80% of lesions occur on the head and neck, while around 15% develop on the shoulders, chest, or back [24]. BCCs are more prevalent in males and are likely attributed to increased exposure to UV light in occupational and recreational settings. There is considerable variation in the BCC clinical presentation. It can appear as an erythematous plaque with obvious telangiectasia, a partially cystic nodule, a papulonodular lesion with a pearly translucent edge, an ulcerated destructive lesion (rodent ulcer), or a pale plaque with variable induration [25]. While the majority of BCCs exhibit slow growth and are relatively non-aggressive tumors, a small percentage display aggressive behavior, leading to the destruction of local tissue and, in rare cases, metastasis [26]. Basosquamous carcinoma is a rare and aggressive variant; it has higher chances for metastatic spread than other basal cell carcinoma forms. The various morphological subtypes of BCC include superficial, multifocal, pigmented, solid (nodular), micronodular, adenoid, cystic, sclerosing, infiltrating, infundibulocystic, keratotic, basosquamous, metatypical, and fibroepitheliomatous BCCs. Numerous histopathological subtypes of BCCs have been identified due to the considerable variation in their morphology. BCCs comprise islands or nests of basaloid cells, with the cells in the centers of the islands arranged haphazardly, and the cells at the periphery, palisaded. Most cases exhibit some relationship to the epidermis's underside. A recently formed stroma distinct from the surrounding dermis covers islands of tumor cells. Usually, there is a variable inflammatory infiltrate.

The cornerstone of managing NMSCs involves surgically removing them through various methods, including conventional surgical excision, non-excisional ablative approaches, and Mohs micrographic surgery. Desiccation and curettage represent among the most commonly employed surgical procedures for treating BCC [27]. Cryotherapy is another treatment approach used for BCC, and it has proven effective, especially in challenging locations like the eyelid [28]. Imiquimod 5% has proven to be an effective topical treatment for several NMSCs involving BCC and Bowen's disease. BCCs and actinic keratoses have been treated with 5-fluorouracil. It has been reported that genital Bowenoid papulosis can be successfully treated with topical tazarotene gel (0.05%). Radiation and photodynamic therapy (PDT) are examples of physical therapies that exhibit potential efficacy.

## Conclusions

We present a series of patients with NMSC. It includes a spectrum of NMSCs highlighting their histopathological and dermoscopic features. Also all the above cases were successfully treated surgically with no evidence of recurrence on follow-up till date, reiterating the role of the dermatologist in detection and treatment of NMSCs. NMSCs are comparatively less dangerous and easily treatable, however, they pose an increased threat due to increased incidence. Knowledge of NMSC pathology and management will help to prevent and treat the disease at the earliest stage.
